# A Green Approach Used for Heavy Metals ‘Phytoremediation’ Via Invasive Plant Species to Mitigate Environmental Pollution: A Review

**DOI:** 10.3390/plants12040725

**Published:** 2023-02-06

**Authors:** Irfan Ullah Khan, Shan-Shan Qi, Farrukh Gul, Sehrish Manan, Justice Kipkorir Rono, Misbah Naz, Xin-Ning Shi, Haiyan Zhang, Zhi-Cong Dai, Dao-Lin Du

**Affiliations:** 1 School of the Environment and Safety Engineering, Jiangsu University, Zhenjiang 212013, China; 2 School of Agricultural Engineering, Jiangsu University, Zhenjiang 212013, China; 3 Biofuels Institute, School of the Environment and Safety Engineering, Jiangsu University, Zhenjiang 212013, China; 4 Department of Biochemistry and Molecular Biology, College of Life Sciences, Nanjing Agricultural University, Nanjing 210095, China; 5 School of Inspection and Testing Certificate, Changzhou Vocational Institute Engineering, Changzhou 213164, China; 6 Jiangsu Collaborative Innovation Center of Technology and Material of Water Treatment, Suzhou University of Science and Technology, Suzhou 215009, China

**Keywords:** invasive plant, biotechnology approaches, heavy metals, detoxification, phytoremediation, hyperaccumulator

## Abstract

Heavy metals (HMs) normally occur in nature and are rapidly released into ecosystems by anthropogenic activities, leading to a series of threats to plant productivity as well as human health. Phytoremediation is a clean, eco-friendly, and cost-effective method for reducing soil toxicity, particularly in weedy plants (invasive plant species (IPS)). This method provides a favorable tool for HM hyperaccumulation using invasive plants. Improving the phytoremediation strategy requires a profound knowledge of HM uptake and translocation as well as the development of resistance or tolerance to HMs. This review describes a comprehensive mechanism of uptake and translocation of HMs and their subsequent detoxification with the IPS via phytoremediation. Additionally, the improvement of phytoremediation through advanced biotechnological strategies, including genetic engineering, nanoparticles, microorganisms, *CRISPR-Cas9*, and protein basis, is discussed. In summary, this appraisal will provide a new platform for the uptake, translocation, and detoxification of HMs via the phytoremediation process of the IPS.

## 1. Introduction

Environments polluted by contaminants produced by anthropogenic activity (mining, petroleum, agro-spray, and fertilization) cause a negative effect on the biosphere as well as on human beings [1]. Two types of contaminants are present, organic contaminants (hydrocarbon, petroleum, pharmaceuticals, pesticides, and alkane) and inorganic contaminants (heavy metals (HMs)) that pollute the environment [2]. HMs generally refer to metals with relatively high atomic weights in the range of 63.5–200.6 g mol^−1^ and densities of more than 5 g cm^−3^. Growing industrial development has increased the level of HMs in ecosystems, causing biological, horticultural, and human problems [3]. Heavy metal pollution is present throughout the ecosystem and is commonly found in industrial areas and near mines [4]. According to Hu et al. [5], twenty million hectares of land worldwide are polluted by HMs. The China National Survey (2005–2013) showed that approximately 19% of investigated land (6.3 × 10^6^ km^2^) was contaminated with toxic HMs, which are mostly Cd, Mn, Pb, Hg, Cu, and Cr [6]. Another study sampled 131 farmland soils in China and found that approximately 6%, 9%, and 6% of samples exceeded Cd, Cu, and Ni limits, respectively [7].

In contrast, some HMs like Cu, Mn, and Zn are required for plant growth in minor quantities and are known as essential metals, but these metals are toxic at elevated concentrations. HMs affect plant growth, reduce physiological and morphological responses, disrupt nutrient uptake activity, and lower plant yield [8,9]. Considering the harmful effects of HMs on living organisms, scientists should control the harmful effect of heavy metals through effective approaches. The physicochemical approach is used to eliminate the toxic metals from the land but is destructive in nature, highly expensive, and disturbs agricultural land, causing secondary pollution [10]. Recently, bioremediation strategies have been used to clean up the environment from contaminants [11]. Bioremediation consists of subtypes, including bioleaching, biofiltration, biostimulation, and phytoremediation [12]. Phytoremediation is one of the bioremediation processes to alleviate toxic contaminants with the help of plants and is low-cost, eco-friendly, and more efficient [13]. Plants are the most effective approach to removing HMs [14]. In particular, weedy plants can carry out phytoremediation to minimize the impact of pollution on ecosystems [15]. Weeds have special attributes, such as absorbing more HMs and storing them in the vacuole to minimize the toxic effects on plants [16]. Weeds grow fast and possess long root systems to enhance metal absorption; hence, they are useful to remediate the environment from toxic HMs [17]. Invasive or native weeds are also reported to minimize the toxic effects of HMs in the environment through various biotechnological strategies [18].

Apart from the native plants, invasive plant species (IPS) and plants transferred from another place are easily spread by air, insects, and water [19]. The IPS damage the diversity of native plants as they spread very quickly and dominantly [20]. The IPS occupy a broad range of biological niches and have unique functional characteristics to rapidly adapt to climatic variation [21]. According to recent estimates, there are more than 232 IPS present in China, and 480 are casual alien species [22]. Approximately 56% of all alien plant species were introduced to China for ornamental purposes, while the remaining 55% were introduced for other purposes, such as medicine, timber, and foraging [22,23,24]. Many articles have reported on the use of various weed plants such as *Calotropis gigantea*, *sida cardifolia*, *Ricinus communis*, *Spartina alterniflora*, *Alternanthera philoxeroides*, and *Eichhornia crassipes* for phytoremediation [25]. Some invasive weeds have shown negative effects on the growth of native plant species but have also demonstrated better growth in contaminated environments and, thus, can be used to clean up agricultural land. IPS can restore the land and have rapid development, strong tolerance, and high adaptation in contaminated areas to support ecological changes. However, very little research has been published about the correlation between IPS and HMs [26].

This review aims to identify and describe the positive correlation between IPS and HMs to emphasize the useful role of invasive plants in controlling heavy metal toxicity through phytoremediation technology. It summarizes the obtainable data on the mechanisms of heavy metal uptake, translocation, accumulation, noxiousness, and tolerance in IPS to determine suitable approaches to improve the efficiency of invasive-weed-based phytoremediation. Additionally, recent biotechnology processes (Genomic editing base, proteomic, Nanoparticles and *CRISPR/Cas-9*) can potentially enhance the heavy metal tolerance capability by using invasive weeds for phytoremediation.

## 2. Uptake, Translocation, and Toxicity of Non-Essential Heavy Metals/Metalloids

Rapid development and urbanization around the world have increased the risk of HMs in ecosystems [27]. The increasing content of HMs in the soils is not only a risk for plant growth and productivity but also threatens human and animal health [4]. Two types of HMs are present in nature, essential HMs (Zn, Cu, Fe, and Mn), which are vital for plant growth and development, but excessive concentrations of these HMs in the soil reduce crop production [28,29]. Non-essential HMs (Cd, Hg, Pb) do not have any known biological function for the agricultural system [4]. HMs adversely affect plant growth by decreasing chlorophyll content and inducing chlorosis and necrosis, and plants finally die due to decreasing chlorophyll content and stomatal closure [30,31]. Moreover, the accumulation of these toxic elements in crops from polluted water, contaminated soils, and agro-spray cause toxic effects on humans (e.g., cancer and kidney disease) [27]. Plants have special membrane transport proteins that absorb essential and non-essential HMs. For instance, Cd and Ca have transporters for entering plants like *IRT*, *NRAMP*, Cu-transporter, *ZRT*, low-affinity divalent ion transporter, and aquaporin transporters. After entry of HMs into a plant, other types of transporters such as P1Btype ATPase, *HMT*, *NRAMP*, and cation diffusion transporters become present in vacuoles, Golgi bodies, and endoplasmic reticula for the transfer of heavy metals from roots to shoots [32,33]. After that, phytochelatins (*PCs*), heavy metals isoprenylated plant proteins (*HIPPs*), metallothioneins (*MTs*), organic molecules, ligands, and compartmentalization are responsible for detoxifying or minimizing the toxicity of HMs [1,15,27]. Some of the non-essential HMs/metalloids, their uptake, toxic effects, and translocation are discussed.

Cadmium (Cd) is a highly toxic heavy metal that affects humans, land, and marine species [34]. When the number of industries and factories increases in the environment, then the concentration of Cd in the land also increases [8]. Cadmium enters the ecosystems through human anthropogenic activities (chemical industries, pesticide spray, and mining) [35]. The accumulation of Cd in the environment is a severe concern due to mobility in the polluted soils and the associated health threats to animals, humans, and plants [1]. In humans, Cd toxicity is shown in the liver, kidneys, and bones and minimizes the body’s ability to absorb calcium [36]. Cadmium is easily absorbed by the crops from the underground water and translocated to the shoot via the phloem with the help of different transporters. It is taken from the soils through the roots using transporters like rice *OsNRAMP* family, *OsIRT1*, and Arabidopsis *AtIRT1* [2,4]. Thereafter, it is translocated to other tissues via phloem with special transporters known as ATPase, such as *AtHMA4* and *AtHMA2* that translocate Cd and Zn from root to shoots, while *OsLCT1* translocate Cd from shoots to other tissues [37,38]. Cd seriously affects plant growth in physiological, phenotypic, and biological terms [27]. Cd has a negative impact on plant productivity by reducing root tips, seedling length, and biomass [39]. Cd toxicity (0.06 g/kg) in soil has been reported to reduce potato crop production and biomass [40]. Cd also affects the physiological response, i.e., it decreases the chlorophyll contents, promotes oxidative stress (ROS), and damages plant membranes and cell macromolecules [35]. Cd also inhibits the uptake of minerals in plants causing leaf chlorosis and necrosis [41]. Cd obstructs the transportation and uptake activity of some essential minerals in plants [42]. Jinadasa et al. [43] observed that Cd affected the cauliflower plant, leading to leaf deficiency symptoms and reduced plant length. Similar results have also been reported in lettuce, radish, and soybeans [14]. Currently, the major concern is reported worldwide; various studies observed the toxic effect of Cd on crop yields, ROS generation, and oxidative damage [27]. The toxic effects of Cd in plants in terms of chlorophyll content, growth development, seedling germinations, enzymatic activities, nutrient uptake, ROS, plant water ratios, and crop yield are further documented [44].

Lead (Pb) is the most common toxic metal after Arsenic (As) and has no known beneficial effect on living organisms [45]. It is emitted from battery preparation factories, fertilizers, insecticide-producing industries, and automakers. Lead also harms wetlands and agricultural land. Root uptake of Pb into plants is accompanied by calcium absorption and adversely affects plant morphology and growth. Upon entering the plant tissues, Pb can disrupt enzymatic activity and hormonal balance, leading to altered nutrient and water uptake, slow growth, and necrotic lesions [46]. The growth of *Spartiana alterniflora* and *Pinus pinaster* was inhibited by Pb [47]. Increased Pb levels in the soils enhance its absorption and negatively impact the plants by increasing ROS and peroxide to damage the enzyme activity [48]. Lead (Pb) poisoning induces reactive damage to plants by the formation of hydroxide ions [49]. Lead toxicity lowers plant development, seed growth, shoot length, biomass production, and enzymatic activity [50]. Lead (Pb) reduces the crop’s biological efficiency in the phenotypical, physiological, and biological processes, as reported in rice at different phases of growth and development [51].

Mercury (Hg) has no beneficial effects on living organisms and is thus considered a primary hazard as it is extremely detrimental to flora, fauna, and agricultural land [28]. Mercury is not easily degraded in the environment and thus is considered a mobile contaminant that affects the ecosystem [52]. Even in small quantities, Hg is hazardous to plants, causing stunted growth and other concerns [53]. It is released into the environment through anthropogenic activities and via natural sources such as volcanoes and is then spread through the air, soil, and water [54]. Organic and inorganic Hg uptake by the plants from the soils is limited, and transfer from the root system to the shoot is also limited. Hg in the soil surface predominantly comes from the environment, while Hg in the rhizome comes from the soil [32]. Kim et al.’s [55] findings revealed that glutathione (GSH) has the potential to confer Hg tolerance by inhibiting Hg accumulation in plants. Ren et al. [56] experimentally showed that together with As, Hg severely affected rice seedlings. The toxic effect of Hg on plant systems varies and includes growth retardation, decreased chlorophyll content, protein denaturation, chlorosis, production of ROS, and DNA damage [53,57].

Nickel (Ni) is a mineral required for plant growth; however, a plant requires a low concentration (0.05 to 10 mg kg^−1^ DW) of Ni, and when Ni’s concentration increases in the environment, it becomes toxic to plant growth and development [58]. When Ni concentration in plants is elevated, it decreases plant growth, increases leaf senescence, decreases nitrogen uptake, and disturbs the Fe level [59]. The Ni concentration in soil depends upon the environment; however, the average Ni content in soil is more than 20 mg kg^−1^ [60]. The content of Ni^2+^ in contaminated soils is 20 to 30 times higher than in native soils [6]. Anthropogenic activities like mining, chemical emissions, coal and oil combustion, wastewater, phosphate fertilization, and insecticides are the boosting sources of Ni^2+^ concentrations [61]. The Ni uptake in the plant system through roots is through active transport or via passive diffusion [62]. After root absorption, Ni is translocated to shoots and leaves through the xylem loading process [63]. The uptake and translocation activity of the Ni pollutant is an active and passive mechanism in plants, and it possesses various toxic effects on plants, such as inhibiting enzymatic activity, degrading proteins, and damaging DNA.

Arsenic (As) is a hazardous metalloid that is toxic, and its concentration in the soil rises from anthropogenic activity (e.g., mining) or naturally occurring (biogeochemical) processes [64,65]. The concentration of As in the soil depends on the environment and occurs in various oxidation states [66]. Both organic and inorganic forms are present in the environment, but the most hazardous and soluble form is inorganic As^3+^, which damages plant growth [67]. As uptake from soils depends on availability, mostly inorganic form (As^3+^, As^5+^) enters the plant tissues via transporters [68]. Once it enters a plant, the plant exhibits various biological, physical, and morphological changes. The absorption of As in plants is difficult to control as it is often controlled by key element transporters [69]. Exposure to As impairs the physiological response (chlorosis), phenotype (growth inhibition), and metabolic activities [70,71]. The most toxic effects of As on plant growth include the production of ROS, denaturation of proteins, chlorosis, necrosis, and disturbance of useful mineral uptake, which sometimes leads to plant death [72].

**Figure 1:**
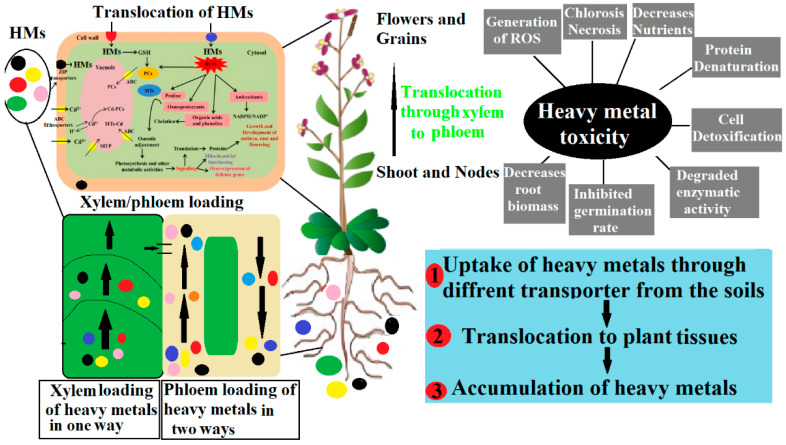
Schematic view of heavy metal (HM) uptake, its translocation towards aerial parts via xylem loading, and its toxicity. The uptake and transport of HMs from the roots to the leaves of plants through apoplastic and symplastic pathways. First HMs enter the plant with the help of various transporters in the roots and are then translocated to different parts. Excessive accumulation of HMs in plant tissues contributes to toxicity with associated effects such as chlorosis, protein denaturation, and decreased plant biomass.

Chromium (Cr), a naturally existing heavy metal used in industrial processes, is present in the earth’s crust and the ocean. Chromium hexa (Cr^6+^) and trivalent (Cr^3+^) are the common oxidation states of chromium, which belongs to the transition metals. Cr^6+^ is more hazardous than Cr^3+^ but less volatile and transportable [73]. The absorption of Cr in plants from the soil can decrease the plants’ physiological characteristics [66]. The exact mechanism of Cr absorption and translocation has not been verified. However, it is absorbed by roots when they form complexes with organic acids [74]. Chromium is poorly transferred to other tissues of the plant after absorption by the root systems [75]. *Pisum sativum* (L.) grown in a potassium dichromate-containing mixture resulted in an increased Cr concentration in different plant tissues [76]. The Cr affects plant growth at the germination stage as well as affects the growth of roots, stems, and leaves, which may impair the total dry matter and decrease the plant’s physiological activities [77] (Figure 1).

## 3. Correlation of HMs and Invasive Plant Species

The global geographic distribution of plant communities, particularly IPS, is under serious threat due to the fragility of aquatic ecosystems [78]. The IPS are frequently dominant over the native ecological areas and disrupt the biome biodiversity [79]. Plant invasion is gaining popularity as it can have a wide range of ecological implications in invaded environments [80]. Most IPS have a set of favorable features that might enable them to quickly become established in non-native habitats [81]. However, the IPS increase the bioaccumulation of HMs in the tissues and release these metals in the form of an organic compound during decomposition [82]. IPS absorb the toxic metals from the soil with the help of the rhizosphere and immobilize these toxic metals, which can significantly reduce the concentration in the soil [83]. The IPS have a strong symbiotic microorganism in the roots that correlate with heavy metals to minimize the toxic effects [84]. Sun et al. [82] reported on many strategies to eliminate toxic heavy metals through immobilization, leaching, decomposition, degradation, and providing the comprehensive biogeochemical behavior of IPS. It has been reported that most of the IPS detoxify environmental HMs with chemical pretreatment, such as NaCl combined with HCl, thereby reducing the toxic effect of Cr and Pb in *Acorus calamus* [85]. *Ageratina adenophora* (*synonym Eupatorium adenophorum*) is one of the IPS reported as a Pb bio-sorbent to adsorb high concentrations and minimize the toxicity of Pb in environments [86]. The interaction of HMs with IPS has a great co-symbiosis process, such as when the invasive weed *Solidago canadensis* was cultivated together with the native plant *Kummerowia striata* in a rich Pb-contaminated area to check its tolerance capability. The results showed that the *S. canadenesis* accumulated more Pb to sequester in aboveground tissues [87]. Wei et al. [88] reported that *Praxelis clematidea, Bidens, Chromolaen aodorata* (*Chromolaena*), and *pilosa* (*Xenarthra*) correlated with Cd and proved experimentally that the invasive *C. odorata* was a possible candidate for a Cd-hyper accumulator to remove Cd through remediation strategies. *Undaria pinnatifida* is one of the invading marine weeds reported to detoxify Hg using calcium chloride to clean up marine environments [89]. IPS that grow fast and have long root systems can detoxify toxic heavy metals in the environment [90]. Hyperaccumulators are plants that collect high quantities of HMs in their aerial organs without exhibiting major phytotoxic consequences [91]. Over 450 plant species have been recorded worldwide to uptake and translocate HMs, many of which are IPS (*C. odorata*, *B. Pilosa*, and *P. clematidea*), making them suitable for phytoremediation [91,92,93]. The invasive plant *Crofton* weeds showed greater hyperaccumulator abilities and tolerance to Cd stress. Additionally, these weeds have a high capability to control pathogen attacks [78]. Invasive weeds interact with the soil microbiome to enhance or tolerate the toxicity of HMs [94]. Most of the IPS are hyperaccumulators to clean up soils contaminated with HMs [14]. The IPS with the potential to clean up the HMs are shown in Table 1.

## 4. Cleanup Environment from HMs by IPS through Phytoremediation

**Figure 2:**
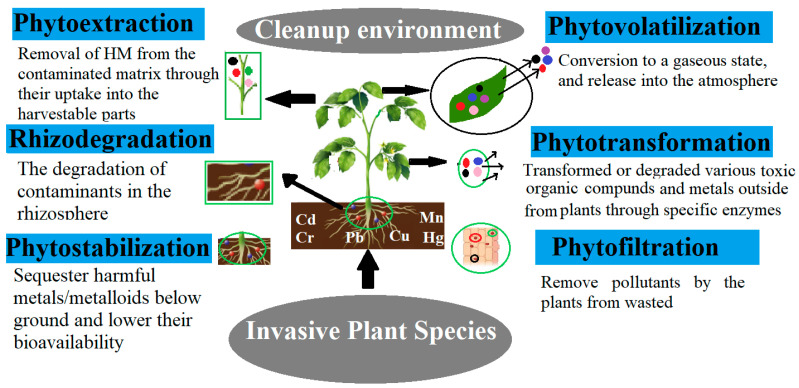
IPS can clean up the HMs through phytoremediation Strategies. Different techniques control or minimize the toxicity of HMs in plants.

Industrial development, current agricultural practices, and other anthropogenic events have increased the abundance of severely toxic HMs in the atmosphere [112]. Overpopulation has caused a surge in industrialization, which in turn has released a lot of pollutants into the environment [16]. Both industrialized and developing nations are seriously concerned about the pollution caused by mining-related activities [113,114]. The development and exploitation of metallurgical resources stimulate the formation of hazardous residues known as mine tailings, which contain potentially toxic elements (PTEs) that may have harmful impacts on the environment [115]. HMs’ restricted mobility affects plant development [116]. Several plants have distinct ways to withstand, neutralize, immobilize, or reverse the harmful effects of HMs, suggesting that certain plant species can establish themselves in polluted sites without alteration [51]. Due to their great adaptability and hyperaccumulation capability, IPS have also been employed to remediate contaminants. Phytoremediation uses IPS to remove environmental pollutants from contaminated soil, sedimentation, sludge, and water. This will be a new insight into the use of IPS as renewable technologies that are low-cost, effective, and eco-friendly to clean up the environment of toxic pollutants. Phytoremediation methods include phytoextraction, phytovolatilization, phytostabilization, phytofiltration, phytodegradation, and rhizodegradation (Figure 2). Most of the plant families Asteraceae, Fabaceae, Poaceae, Violaceae, and Lamiaceae can perform phytoremediation, and most of the IPS belong to these families [11]. At present, this technique is used worldwide, especially in China, and particularly in those areas where more toxic HMs are present, like Cd, Cu, Pb, and As [110]. Heavy metal hyperaccumulators have shown promise in phytoremediation, and about 450 heavy metal hyperaccumulator plant species have been documented globally. However, not all plant species are possibly valuable for phytoremediation in contaminated sites, as they require rapid growth and large root systems [117]. Pontederiaceae, Asteraceae, and Fabaceae are the most representative plant families with a large number of species that bio-accumulate HMs [118]. Furthermore, recent studies have shown that the IPS can be used as biosensors for heavy metal-contaminated sites, such as *Tamarix tetrandra Pall*, *Alliaria petiolata* (*M. bieb*), *Robinia pseudoacacia* L., *Miscanthus*, *Amorpha fruticosa* L., and *Populus alba* L., and can eliminate different HMs with phytoremediation [119,120]. Wei et al. [88] experimentally proved that *C. odorata*, *B. pilosa*, and *P. clematidea* enhance tolerance to Cd stress and have more ability to phytoextract Cd without morphological and physiological disturbance. Some of the invasive weeds can destroy toxic elements such as Cd, As, Hg, and Pb at specific temperatures [18]. Several researchers have also demonstrated that most invasive weeds can help squeeze the contaminants from polluted areas to clean the environment [14].

### 4.1. Tolerance/Detoxification Mechanism of IPS against HMs

Plant normal growth and metabolic activities use two approaches to detoxify HMs: avoidance and tolerance. The avoidance approach is also known as a first defense mechanism because the plants absorb fewer numbers of HMs through roots, which limits the movement to the upper part of the plants [121]. Root sorption, metal precipitation, and metal exclusion are the defensive mechanisms of the avoidance process. In these defensive mechanisms, root sorption (root exudates) acts as a ligand to form complexes with HMs to decrease the availability and toxicity of HMs, while metal precipitation inhibits HM transfer from the soil to the roots/shoots through adsorption, absorption, and chelation processes [122]. The metal exclusion mechanism works in between the root and shoot systems as a defensive mechanism against HMs to reduce toxicity. For example, arbuscular mycorrhizas can inhibit the HM provision into the roots through a process of absorption, adsorption, and chelation. On the other hand, the second tolerance approach is initiated when HM ions enter the cytosol, after which the plants detoxify the HMs through chelation/compartmentalization in certain cellular organelles, such as the vacuole, where HMs are sequestered by complexation with transport proteins, including metallothionein (MT) and phytochelatins (PC) [121,122].

IPS have a potential for detoxification/tolerance of HMs via the phytoremediation process [123]. For example, *Bromus tectorum* is an IPS and a better option for a hyperaccumulator due to its high nitrogen-absorbing activity and tolerance to graminicides and metals [124]. The allelopathy of *S. canadensis* and *C. canadensis* enhance the tolerance of the indigenous *Lactuca sativa* (L.) against Cu and Pb [125]. A comparative study of a native plant (*K. striata*) and an invasive plant (*S. canadensis)* reported different concentrations of Pb(AC)_3_. 3H_2_O. The findings suggested that the rapid growth of *S. canadensis* in Pb(AC)_3_. 3H_2_O-contaminated soil compared to *K. striata* was due to its ability to eliminate or reduce Pb uptake [94,126]. *P. stratiotes*, a fresh-water invasive species, has been approved to remove various pollutants from dirty water with the help of phytoremediation techniques (Phyto-filtration lagoon) [127]. *P. australis* is one of the IPS in America that immobilizes Hg concentration inside the roots [128].

### 4.2. Elimination of Toxic HMs via IPS

Recently, several methodologies, including chemical precipitation, ionic exchange, membrane filtration, and ultrafiltration, have been used for the treatment of HM pollution [129]. Unfortunately, these methods have some drawbacks, including high costs, time-consuming procedures, and complex operations. Adsorption is one of the defensive mechanisms of IPS for HMs detoxification and is highly recommended because it offers a wide range of advantages, such as eco-friendliness, high performance, and simplicity in its procedure [130]. The use of invasive plants as biosorbents for the removal of heavy metal ions may meet these critical requirements. IPS nowadays are used to clean up contaminated lands and ecosystems and eliminate toxic HMs with the help of remediation strategies [131]. *Lolium multiflorum* has a potential tolerance to Cd as a hyperaccumulator capability [132]. Sanghamitra et al. [133] observed that the invasive plant *Parthenium hysterophorus* L., belonging to the family Asteraceae, was a beneficial species for zinc hyperaccumulation; it removed the toxic effect of Zn in soil and cleaned up HMs. *Lythrum salicaria*, an invasive plant abundantly found in the USA, is a perfect example of remediation of Pb-polluted environments to clean the Pb concentration in the soils [134]. *Pluchea sagittalis* (L.) is an example of an invasive anti-Pb biological-defense mechanism to squeeze Pb concentration [135]. The strong clonal-integration capacity allows IPS to disseminate from terrestrial into aquatic biotopes stressed by HMs, and such IPS have the tendency to sustain vegetation in aquatic biotopes degraded by HM pollution [136].

### 4.3. Phytostabilization

Phytostabilization is a process occurring through different mechanisms, including precipitation, sorption, complexation, and metal valance reduction in the rhizosphere of plant roots for erosion, leaching or run-off prevention, and the conversion of HMs [137]. Such an HM conversion can be seen in the classic case of the reduction of Cr^+6^, a more toxic form of this metal, to Cr^+3^, a more mobile and less toxic species [138]. Commonly, this process is used to stabilize the toxic effect of HMs in contaminated water, soil, sediment, or sludge, preventing their migration to groundwater or their entry into the food chain [139]. Rhizosphere sorption in plant roots, soil additives, chelation, and deposition are biological methods of phytostabilization for immobilizing toxic metals from one place to another [140]. Phytostabilization is also known as phytoimmobilization because several plant root exudates are bound with toxic HMs, which convert non-toxic form to ecosystems [141]. HMs conjugated with derivatives of amino acids, proteins, and sugars form complexes in the rhizosphere to immobilize the toxicity of toxic metals. For example, the As toxic form converts to a non-toxic form (As-tris-thiolate) after it forms a complex with ferric sulfate in the rhizosphere within the vacuoles [142]. Usman et al. [143] revealed the phytostabilization of an invasive plant *(P. juliflora)* in primary growth stages on metalliferous soils, which had the capability to clean up Pb and Cd. Some of the IPS are employed as biotechnological tools for bioremediation [144]. *P. glandulosa* is a kind of IPS that can phytostabilize toxic metals and clean up the environment [145].

### 4.4. Phytoextraction

Phytoextraction is the part of phytoremediation to uptake HMs from the soil through the roots and translocate them to above-ground parts, allowing for more accumulation of HMs without any harmful effect. This is the permanent solution for HMs detoxification through the harvesting biomass. Such processes are also called phytoabsorption, phytoaccumulation, and phytosequestration because of the absorption of various toxic metals through roots from the soil or water, which are then accumulated in the upper parts of the plants to be eliminated through the harvesting of the biomass [142]. Phytoextraction eliminates various toxic heavy metals from the ecosystem through various plant species, especially those which grow very fast and are deeply rooted, such as IPS [19]. There are two concepts of phytoextraction, (1) continuous phytoextraction, which is a natural phenomenon present inside plant species to store more HMs without causing any effect (hyperaccumulator), and (2) induced phytoextraction, where additional components are added to the plant body to control the effect of the HMs by means of a phytochelatin-formed complex, which translocates to the vacuole, cell membrane, and other metabolically inactive parts [140,146].

According to Begonia et al. [147], plants with rapid proliferation and a stronger root system to improve phytoextraction, such as Coffee weed (*Sesbania exaltata*), are effective at eliminating Pb from polluted soil. Yang et al. [148] also investigated the capability of three cultivars of Napier grass (*Pennisetum purpureum*) for absorbing Cd and Zn in the field and discovered that *P. purpureum* cv. *Guiminyin* collected the highest levels of Cd and Zn in its shoots. *P. stratiotes*, often known as water lettuce, is one of the IPS that has been widely used due to its potential for the hyperaccumulation of HMs [149]. “*Eupatorium adenophorum*” is an invasive species that produces biochar to remove HMs from contaminated areas and possesses a high adsorption capacity [150]. The adsorption capacity of *S. alterniflora* showed an increased removal of Cu ions from the soil and freed the land from pollutants [151]. “*Ipomoea carnea*” is one of the Indian invasive species and has a great capability to accumulate more Cd, Cu, Cr, and Pb without any harmful effect on the plants [152].

### 4.5. Phytodegradation

Phytodegradation or phytotransformation transforms or degrades various toxic metals into less toxic forms using an internal plant metabolic process or an external plant body process, known as rhizodegradation, through specific enzymes produced in the roots, such as dehydrogenase, peroxidase, oxidoreductase, and dehalogenase. For instance, *Liriodendron tulipifera* is grown among highly toxic concentrations of Hg^2+^, and then these toxic metals are transformed into a less toxic ionic form of Hg. From this point of view, the green plant, also known as “green liver,” is vital for the removal of HMs in the biosphere [137,153]. Hannink et al. [154] reported that tobacco roots generated the nitro reductase enzyme (NfsI), which helped to degrade TNT (trinitrotoluene) in the soils. *P. deltoids* plants converted RDX (hexahydro-1,3,5-trinitro-1,3,5-triazine) to physiological components and decreased its toxicity [155]. Recently, Papadopoulos and Zalidis [156] conducted a trial culture to remediate the terbuthylazine (TER)-contaminated wetland and discovered that the root system of *Typha latifolia* was a potential plant for TER phytodegradation. The plants associate with microorganisms to degrade or transform toxic HMs to remediate them through biochemical pathways known as rhizodegradation [157]. A rhizophora mangle mangrove combined with plant growth-promoting rhizobacteria (*Pseudomonas aeruginosa* and *Bacillus* sp.) was able to metabolize polycyclic aromatic hydrocarbons (PAHs) in contaminated soil [158]. *A. philoxeroides* is one of the invasive weeds that can potentially be used for phytoremediation to degrade the sulfonated azo dye Remazol Red (RR) [17].

### 4.6. Phytofiltration

Plant roots, shoots, and seedlings are used for the removal of pollutants from a waste area or contaminated water in a process known as phytofiltration [159]. In this process, contaminants are absorbed or adsorbed from contaminated surface waters or wastewater, restricting their movement in underground waters. This strategy may be conducted in situ, where plants are grown directly in the contaminated water body, decreasing costs. Gomes et al. [160] described three types of phytofiltration, known as rhizofiltration (roots), caulofiltration (shoots), and blastofiltration (seedlings). During the rhizofiltration, plant roots were used to absorb the pollutants from the polluted soils and water to clean up the environment by means of accumulation, adsorption, absorption, and precipitation into plant biomass [161].

Both terrestrial and fast-growing aquatic plants can be used in rhizofiltration to extract Cd, Cr, Cu, Ni, Pb, and Zn. In biogas production, a phytofiltration lagoon containing the *P. stratiotes* invasive species is particularly feasible for delivering biomass all year and successfully treating dirty water [162]. The water hyacinth could be a viable, cost-effective, and environmentally friendly option for the phytoremediation of pesticide-contaminated agro-industrial wastewater [163]. *E. crassipes*, *P. stratiotes*, and *M. spicatum*, three invasive aquatic plants, were discovered to be viable methods for in situ cleanups of heavily contaminated rural rivers in developing nations, including China [109]. Rhizofiltration can be used to clean up the environment from toxic contaminants using the phytoremediation process of either root absorption or adsorption (Figure 2).

## 5. Biotechnological Processes for Enhancing Phytoremediation Potential of IPS

**Figure 3:**
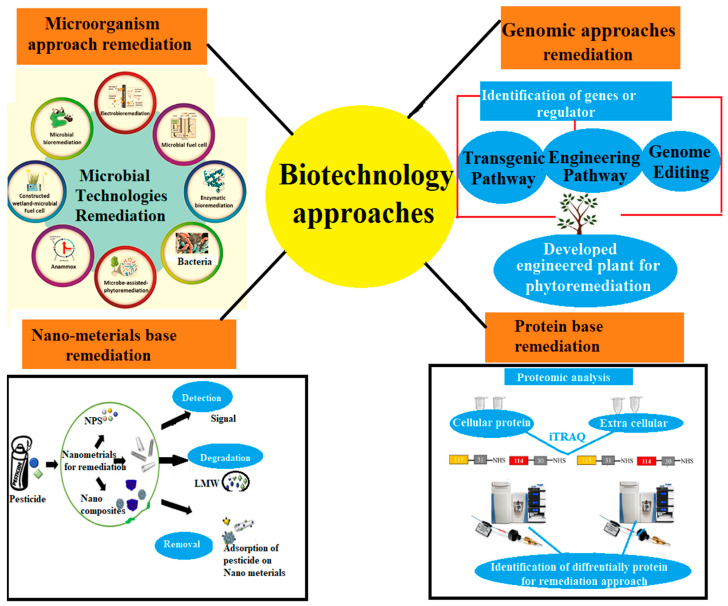
Biotechnology approaches that enhance remediation strategies.

Different techniques and strategies have been verified to enhance plant phytoremediation capabilities against HMs, for example, genomic methods including *CRISPR/CAS9* (Clustered Regularly Interspaced Short Palindromic Repeat-Associated Nuclease 9), proteomic approaches, transcriptomics, metabolomics, and transgenic plant-based, nanoparticle-based, and plant–microbe-based methods [164]. Various techniques have been established to enhance the remediation strategies, with some of them discussed below (Figure 3).

### 5.1. Genomic Approaches

In the past few decades, genetic modification techniques have been used to improve the performance of plants as cleanup options for HMs [165]. The term “genomics” refers to the study of gene transcriptomics, metabolic engineering, transgenic, gene editing, and proteomics. Plant genomic research has made great strides, enabling the development of a wide variety of vital crops and weeds. The gene insertion method is one of the modern biotechnological approaches used to control HMs in a contaminated environment. A gene is inserted into the plant through a tissue culture or gene gun method, which genetically modifies the plant and increases its capabilities to detoxify or tolerate hazardous metals. This method is based on the insertion of genes into plants with a fast growth rate to promote the tolerance for and hyperaccumulation of toxic HMs [166]. Transgenic plants can potentially increase the phytoremediation processes. For example, in rice, *OsHIPP42* was inserted into rice tissues, and the rice decreased the toxicity of Cd in the environment [27]. A similar study was also reported in a tobacco plant, into which an MT1 gene was inserted, which significantly enhanced the plant’s tolerance capability against Cd and Zn [167]. This method can also be used to boost biomass, metal storage capabilities, and multiple HM hyperaccumulation [168].

The *CRISPR–Cas9* system is a novel gene-editing tool widely utilized for gene knockout research and genome editing in a variety of plant growths. It consists of a Cas9 nuclease that causes a genomic dual break (DSB) and a guide RNA (*gRNA*) that directs movements to a specific location in the genome. *CRISPR*/Cas9 mainly consists of three types: Type I, Type II, and Type III—a simple, convenient, and easy system [169]. *CRISPR* was first reported in 2012 in mammalian cells and in 2013 in plants [169,170]. Due to the excess Guanine Cytosine content in monocotyledon plants, the *CRISPR/Cas* is more valuable [170]. This approach enhances the capability of phytoremediation against different HMs through *CRISPR-Cas9-modified* transgenic plants. Recently *CRISPR*/Cas9-modified plants (transgenic) were used to minimize, immobilize, and sequester the HMs [171]. For example, in *Oryza sativa*, the *CRISPR*/Cas9-modified metallochaperones gene OsHIPP16 decreased the Cd accumulation in the mutant lines and overexpression lines to minimize the toxic effect [172]. Enhancing antioxidant potential is the most prevalent technique for increasing heavy metal tolerance with the help of genetic modification [173]. However, the genetic modification strategy has shown promising results in enhancing bioremediation approaches.

### 5.2. Microorganism-Based Phytoremediation

Microorganism-based technology is used as bioleaching because the microorganisms can penetrate the rhizosphere system and promote plant growth, as well as break down hazardous substances or convert them into less damaging forms. This biotechnological process is more efficient at detoxifying or alleviating contaminants from the soil through various microorganisms. It has been reported that the phytoremediation potential of plants is enhanced by plant-growth-promoting bacteria (PGPB) [174]. HMs are taken up by the roots, and the bacteria are responsible for decontaminating the environment of HMs by secreting several chemical siderophores (chelators) and organic acids [63]. Many PGPBs have been shown to improve the phytoremediation capability of plants by enabling root uptake of HMs. Several other bacterial species have been found to release polymeric substances, such as cellulose and glomalin, which help to stabilize HMs by reducing their mobility [175]. Numerous IPS had major consequences on the biota connected with them in various parts of the world to control the toxicity of HMs [176]. Elsheikh et al. [177] reported that IPS potentially alter the composition and diversity of the aboveground plant community structure. Havryliuk et al. [178] reported that the invasive plant (*S. canadensis*) immobilized the Cu concentration with methane–sulfate-reducing bacteria with the help of phytodegradation. Usually, the viability of invasive exotic plants is influenced by soil microbial populations [179]. Furthermore, soil biota can help spread pathogens in the new lands, and it was proposed that experiencing fewer soil-borne pathogens might be beneficial to control pollution [180]. Some of the bacterial strains in IPS were confirmed to enhance the tolerance of HMs [177]. Endophytic bacteria of IPS help the plant to grow in stressful conditions without any harmful effects. Srivastava and Anandrao [181] identified more than 400 fungal strains in invasive plant (*Prosopis juliflora*) leaves, which help the plant survive in adverse situations such as drought, salt, infections, and environmental toxins. The methagonic microbial preparation significantly enhanced the tolerance of Cu in *P. stratiotes* using anaerobic degradation [182]. IPS (*W. trilobata*) enhanced the phytoremediation with phosphate-solubilizing bacteria in Cu-polluted soils [183].

### 5.3. Nanoparticles-Assisted Phytoremediation

Recently, nanoparticles have been used as a new technique to enhance the tolerance of HMs, and therefore these advanced techniques can also increase the capability of phytoremediation [184]. Nanoparticles are a class of metal compounds that include particulate substances that can help to stabilize the HMs so they can be adsorbed in plants (Table 2). Nanoparticles (NPs) help to increase plant productivity and enhance the removal of environmental pollutants through phytoremediation [100,185]. Cd-polluted soil supplemented with TiO_2_NPs has been reported to improve the growth of soybean (*Glycine max*) plants [31]. The NPs of salicylic acid were added exogenously (SANPs), and the result showed that *Isatis cappadocica* could be improved throughout the early stages of growth [186]. Huang et al. [39] observed that applying a nanotechnology zero-valent iron (nZVI) to *Boehmeria nivea* and *L. perenne* increased the potent antioxidant mechanism and phytoremediation potential of cadmium. The antioxidant response and metallothionein expression levels of *L. luteus* were significantly increased with the provision of silver nanoparticles [187]. Hussain et al. [188] observed increased resistant activity of radish (*Raphanus sativus*) against lead accumulation and enhanced tolerance response when treated with thidiazuron (TDZ) and magnesium oxide (MgO) NPs. Copper nanoparticles are used to alleviate the toxic effect of HMs in cereal crops, such as rice and barley plants, and can induce Cd accumulation after treatment with CuONPs and CuNPs [189].

### 5.4. Protein-Based Phytoremediation

This method is also known as the proteomics approach. Proteomics is a branch of biology that studies the encoded proteins in the living organism that play significant roles in different species through molecular and physiological levels [190]. This biological process acts as a significant pathway for transmitting information about plant growth and adaptation to metal toxicity [191]. Plant circulatory networks, for example, transfer nutrients, water, and harmful ions (Cd) from the roots to the leaves via xylem sap; in this process, the Cd in xylem sap is considerably detoxified due to proteomics [192]. Detoxification of heavy metals uses different defense processes, such as exclusion, compartmentalization, and formed complexes with different proteins, e.g., phytochelatins (PCs) and metallothioneins (MTs), in different plant species [193]. The iTRAQ method was reported to alleviate the Cd in the root of the maize plant [194]. The protein-encoding gene *OsMTP1* increased the tolerance against Cd in the tobacco plant, which is promising for phytoremediation [195]. ABC Shuttle’s glutathione-S-conjugate pumps relatives between hydroxylated glutathione, glutathione-related organic compounds, xenobiotics, and formed peptide-metal complexes with phytochelatins to minimize the toxicity of HMs [196]. Toxic compounds are transported out of the cells or stored in vacuoles for chemical deprivation and transformation into less harmful biological molecules [197]. The active transport of metal ions into the vacuole and chelation via activation of the metal-binding peptide protein formation of a metallic complex contributes to metal detoxification [198]. Metal chelators are proteins with a low molecular weight that have been identified as crucial methods for metal detoxification in plants [199]. Metal chelate complexes, e.g., PCs, amino acids, glutathione, and MTs, play a significant role in controlling metal toxicity [96]. The protein-based phytoremediation has the potential to control the HMs in polluted lands [200]. Biotechnology-based remediation techniques are indicated in Table 2.

**Table 2:** Biotechnology approaches and metal detoxifying mechanism of HMs by invasive and other naturalized plant species.

Species	Metals	Remediation Role	Reference
*O. sativa* *A. thaliana* *S. cerevisiae* *N. tabacum* *N. tabacum* *O. sativa* *C. demer* *sum* *P. aeruginosa* *R. opacus* *B. subtilis* *A. faecalis* *B. cereus* *B. firmus* *P. aeruginosa* *S. cerevisiae* *E. camaldulensis*	Cd Cd Cd Cd, Zn As Cd Cd As, Cu Pb, Cd Hg, Cd Cd Pb Pb, Cu Cr Pb, Cd Cu	*OsHIPP42* gene enhanced Cd flux and detoxification *AtCd19* help to Cd tolerance in *Arabidopsis thaliana* Enhanced Cd tolerance with the help of *TaHIPP1* *ScMTII* enhanced Cd and Zn accumulation Enhanced As phytoaccumulation through *AtACR2* *OsMTP1* help to increase Cd accumulation *CdPCS1* increase accumulation through hyper accumulator Through biosurfactant production with the help of microbes Adsorption in exopolysaccharides of Cd and Pb Increase the Biosorption capability in plants Adsorption and/or precipitation Bioaccumulation and biosorption Adsorption in exopolysaccharides Cr reduction Biosorption Act as an antioxidant. Well-known DEPs photosynthesis, metabolism, transcription, and translation	[27] [201] [7] [202] [203] [204] [205] . [206] [207] [208] [209] [209] [209] [210] [211] [212]
*A. hypogaea*	Cd	various DAPs linked with heavy metal transport to detoxify Cd	[213]
*O. sativa*	As	Deferentially expression proteins decrease As accumulation	[214]
*C. intybus*	Pb	81 DAPs were identified for metal toxicity.	[49]
*D. officinale*	Cd	2469 DEGs identified and helped to decrease the toxicity of Cd	[215]
*P. distichum* *C. tinctorius* *B. napus* *G. max* *T. repens* *B. nivea* *T. aestivum* *V. faba* *I. cappadocica* *P. australis*	Hg Cd Co Cd Sb Cd, Fe Cr, Cd Cd, Pb As Si	Expression patterns of metal binding and transport protein Phytoextraction of Cd with nano-ZnO to remove HMs Decreases the Conc. of Co with nano-Co3O4 NPs TiO2 removed Cd through Phytostabilization Stabilization of Cr(VI) into a less toxic form Squeeze the Cd and availability of Fe through nZVI Enhanced and tolerate Cd and Cr with Fe-NP C-NPs help to immobilize metal-phosphate Stabilization by ion exchange, surface Phytoremediation in estuarine areas	[110] [216] [217] [122] [218] [219] [220] [25] [186] [221]

## 6. Future Perspective

Despite the fact that invasive plants are recognized as a serious risk to the environment and the biosphere, it is clear that their high tolerance, wide distribution, and rapid growth have the potential to be useful in a variety of applications. The IPS have a positive effect on the biosphere because they have strong biomass production and are used in herbal medicine, nourishment, ornaments, biochar production, paper manufacturing, different cosmetics, and livestock feeds [4,18]. IPS are used to produce various nanoparticles, aerogels, biofuels, carbon products, and biogas. Consistent with recycling and reuse initiatives, the bio-waste of invasive plants can be utilized as adsorbent substances for the elimination of contaminants from polluted environments.

To ensure the integrity of biowaste from IPS, more attention needs to be paid to the sustainability of these types of sources. Removing toxic pollutants from the environment with the help of IPS is a great achievement; for this purpose, the development of invasive plants will be required in industrial mining areas.

Nano-remediation is a revolutionary approach that uses the incorporation of microbial cells to improve the effectiveness of eliminating HMs from polluted areas. However, to increase the scope of contaminated soil remediation, the relationship between molecular techniques and nanotechnology must be elucidated.

The strategy to clean up HMs using genetic engineering is promising, but to date, no exact biotechnological approach for HM detoxification has been identified, and there is a lack of the availability of entire genome data in IPS. Therefore, further studies need to be carried out.

## 7. Conclusions

Heavy metal contamination is a serious issue for plants, animals, aquatic life, and human health. Phytoremediation is a green, low-cost, socially acceptable, and ecologically sustainable technique. In this review, we summarized the uptake, translocation, toxicity, and detoxification of HMs from the soil to plants and presented suitable bioremediation strategies. This study also covers the biotechnology tools, such as genetic engineering, CRISPR/Cas9, microorganism-assisted phytosanitation, AMF colonization, and nanoparticles (NPs), that are widely used to enhance phytosanitation. Furthermore, this study discussed the nature of IPS, their correlation with HMs, and heavy metal detoxification with IPS using phytoremediation. According to the literature review, IPS can be considered a possible choice for phytoremediation.

## Figures and Tables

**Table 1:** Correlation of IPS with HMs and appropriate mechanism.

Invasive Species	Interaction with HMs	Processes	References
*A. philoxeroides*	Pb, Cd	Phytostabilization	[48]
*X. strumarium*	Cd, Cr	Hybridization	[95]
*A. philoxeroides*	Cd, Cu, Zn	Tolerance	[25]
*A. retroflexus*	Cu, Pb	Competition	[14]
*F. japonica*	Cd, Zn, Cr, Pb	Tolerance	[96]
*A. adenophora*	Cd, Cr	hyperaccumulator	[78]
*E. crassipes*	Cd	Phytostabilization	[97]
*E. crassipes*	Cd, Hg	Phytofiltration	[98]
*T. latifolia*	Cd, Zn, Cu	Phytoextraction	[99]
*A. spinosus*	Pb and Cd	Phytoextraction	[100]
*I. aquatica*	Pb	Phytofiltration	[101]
*E. canadensis*	Co	Phytofiltration	[102]
*T. diversifolia*	Zn, Cd, Pb	Phytoextraction	[103]
*P. indica*	Pb and Ra	Phytostabilization	[104]
*A. philoxeroides*	Industry dye	Phytodegradation	[105]
*A. artemisiifolia*	As, Cd, Cr, Cu, Mn, Ni, Pb,	Phytoextraction	[106]
*A. donax*	Pb and As	Phytoextraction	[107]
*A. conyzoides*, *B. pilosa*	Zn, Pb, and Cd Cd, Zn	Phytoextraction Phytoextraction	[108] [108]
*C. odorata*	Cd	Phytoextraction	[88]
*E. crassipes*,	Nutrients	Phytofiltration	[109]
*P. distichum.* L	Hg	Phytostabilization	[110]
*P. perfoliatum*	Mn	Phytoextraction	[111]

## Data Availability

There is no data availability statement.
